# Typhoid Fever in the 42d Wis. Infantry, Stationed at Cairo, Ill.

**Published:** 1865-01

**Authors:** Geo. D. Winch

**Affiliations:** Surg. 42d Wis. Infantry


### TYPHOID FEVER IN THE 42D WIS. INFANTRY,
### STATIONED AT CAIRO, ILLINOIS.

By GEO. D. WINCH, Surg. 42d Wis. Inf.

It has been four months since the enlistment of this Regiment, and they have been stationed at Cairo most of the time. The men are mostly farmers, of good constitution.

I first see these cases when brought to the hospital, and on examination discover the following symptoms: They have had one or two days of feeling chilly, very weak in the limbs, with some pain in them. Nearly one quarter of the cases have a severe headache that remains three or four days and then passes gradually away. This symptom does not otherwise change the character of the disease. Pulse averages from 90 to 120, and soft. Frequently no inclination for sleep the first two or three days; often detect tenderness over the abdomen in this early stage. At the expiration of four or five days there are two directions for the disease to run.

One class continues a mild course, pulse never rises above 100, no delirium, tongue moist, secretions of the kidneys and intestines are nearly normal, except a slight alkalinity of the urine, which I will soon notice. If sordes collect on the teeth they will soon abandon their illegitimate situation. After continuing in this condition for two or three weeks, the light abnormal symptoms clear away by degrees and the patient in a very short time is able to be returned to duty.

In the other and more severe grade, the pulse continues to increase in frequency until it rises to 110 or 120, very seldom 130, becoming weak and thread-like; the tongue coated, dark and dry, many cases fissured, and in a still less proportion swollen. The skin of the feet and hands dry and scaly; tremor of the muscles on moving. Extreme deafness in many cases, and frequently a thin discharge from the ears, amounting to three or four ounces in twenty-four hours. Delirium consists of a low, unconscious muttering. In but very few cases flaccitation. Between the sixth and tenth day of the disease a cough commences, accompanied by expectoration. Auscultation detects crepitation with sibilus. These pulmonary symptoms are frequent, and gradually clear away sooner than the other symptoms. Bed-sores have been unusually frequent and severe; the sensibility frequently blunted.

I have closely watched for eruptions in all its stages. The only class I was able to discover was sudamina; they were two lines in diameter, containing a white, thin fluid, had no red bases. By passing the hand over them it produces a feeling of roughness. They are present in one-third of the cases of this kind, commencing near the fifth, and lasting from six to nine days, and then dry away leaving the surface in a scaly powdered condition. The perspiration was more free in the cases with the eruption than those without it. From the 14th to the 20th day, sometimes much later, the symptoms commence improving.

Pulmonary symptoms, which commence after the disease has begun, disappear first. The urine at the commencement gives alkaline reaction to test paper, in nine-tenths of the cases, continues so through the disease, unless changed by the free use of acids. Its specific gravity was found much below that of normal urine in a very few obstinate and long-continued cases. It did not coagulate by the Nitric acid or heating test.

Treated thirty-three cases, lost one. On examination of the abdomen found the small intestines of a slate color; the lower portion of the ilium filled with a dark substance, about the consistence of mucilage, evidently more or less blood; the mucous coat of the whole tract of the ilium was thickened and congested. Ulceration was confined to the last five inches of the ilium, in which were found four ulcers, about three lines in diameter, which had destroyed the mucous coat. Kidneys and liver had a healthy appearance; the omentum healthy; thorax not examined.

Although there is so much liberal writing on the treatment of typhoid fever, it is difficult to find a guide in all cases. In this disease, as in many others, the celebrated prescriptions extolled by distinguished men will not perform the work they are intended to execute, especially among troops that *frequently change their location.* Judging from the alkalinity of the urine; and observing that those patients whose urine gave the strongest alkaline reaction, were the most dilatory in their recovery; and those that had a relapse were the patients that had alkaline urine when they left the hospital, and when they returned, I came to the conclusion that the remedies should be acid, and the practice has decided the virtue of the theory. The cases that come under the first class will do well without much treatment. Give a small dose of some alcoholic stimulant after meals, beef-tea every three or four hours. If necessary, an opiate to quiet wakefulness, and stimulating expectorants for the lung difficulty; also mur. acid, dil., gtts. xv, three times a day.

But the second class, where the urine is obstinately alkaline, and other symptoms severe, require more meddlesome treatment. Give dil. mur. acid, m. viij, as frequently as once in three or four hours, combined with tinc. opii in small doses as nervous symptoms arise. Let the patient occasionally have a drink of solut. bi-tart., chlor., or bi-carb. pot. As pulmonary symptoms exhibit themselves, combine with other remedies, fluid ext. seneka in moderate doses, and here again will the opiate be a useful auxiliary. I find sul. quinia, given in from one to two grain doses, four or five times a day, combined with the other remedies, to be useful. *Give it for its tonic effect.* The mur. tinc. of iron, with tinc. gent. comp., has worked admirably in those cases that were anæmic, not omitting the mur. acid. But I am confirmed that it is not so useful for a tonic, except in *those* cases, as quinine. In a large proportion of cases it has been necessary to move the bowels with gentle cathartics. After a trial of different methods of treatment, I find patients recover in less time, are not as liable to relapse, and the pulmonary symptoms less severe, in those cases where the mineral acids have been used, in combination with the other necessary treatment.

